# Phylogenetic and functional analysis of cyanobacterial Cytochrome *c*_6_-like proteins

**DOI:** 10.3389/fpls.2023.1227492

**Published:** 2023-09-06

**Authors:** Alejandro Torrado, Macarena Iniesta-Pallarés, Adrián Velázquez-Campoy, Consolación Álvarez, Vicente Mariscal, Fernando P. Molina-Heredia

**Affiliations:** ^1^ Instituto de Bioquímica Vegetal y Fotosíntesis (Universidad de Sevilla, Consejo Superior de Investigaciones Científicas), Sevilla, Spain; ^2^ Institute of Biocomputation and Complex Systems Physics, Universidad de Zaragoza, Zaragoza, Spain; ^3^ Departamento de Bioquímica y Biología Molecular y Celular, Universidad de Zaragoza, Zaragoza, Spain; ^4^ Instituto de Investigación Sanitaria Aragón (IIS Aragón), Zaragoza, Spain; ^5^ Centro de Investigación Biomédica en Red en el Área Temática de Enfermedades Hepáticas y Digestivas (CIBERehd), Madrid, Spain

**Keywords:** cytochrome *c*_6_, cytochrome *c*_6_-like proteins, cytochrome *c* oxidase, cyanobacteria, cytochrome *b*_6_ *f* complex, photosynthesis, respiration, electron transfer

## Abstract

All known photosynthetic cyanobacteria carry a cytochrome *c*_6_ protein that acts transferring electrons from cytochrome *b*_6_ *f* complex to photosystem I, in photosynthesis, or cytochrome *c* oxidase, in respiration. In most of the cyanobacteria, at least one homologue to cytochrome *c*_6_ is found, the so-called cytochrome *c*_6B_ or cytochrome *c*_6C_. However, the function of these cytochrome *c*_6_-like proteins is still unknown. Recently, it has been proposed a common origin of these proteins as well as the reclassification of the cytochrome *c*_6C_ group as *c*_6B_, renaming the new joint group as cytochrome *c*_6BC_. Another homologue to cytochrome *c*_6_ has not been classified yet, the formerly called cytochrome *c*_6-3_, which is present in the heterocyst-forming filamentous cyanobacteria *Nostoc* sp. PCC 7119. In this work, we propose the inclusion of this group as an independent group in the genealogy of cytochrome *c*_6_-like proteins with significant differences from cytochrome *c*_6_ and cytochrome *c*_6BC_, with the proposed name cytochrome *c*_6D_. To support this proposal, new data about phylogeny, genome localisation and functional properties of cytochrome *c*_6_-like proteins is provided. Also, we have analysed the interaction of cytochrome *c*_6_-like proteins with cytochrome *f* by isothermal titration calorimetry and by molecular docking, concluding that *c*_6_-like proteins could interact with cytochrome *b*_6_ *f* complex in a similar fashion as cytochrome *c*_6_. Finally, we have analysed the reactivity of cytochrome *c*_6_-like proteins with membranes enriched in terminal oxidases of cyanobacteria by oxygen uptake experiments, concluding that cytochrome *c*_6D_ is able to react with the specific copper-oxidase of the heterocysts, the cytochrome *c* oxidase 2.

## Introduction

Oxygenic photosynthesis is the major biochemical reaction that facilitates life on earth (Falkowski and Isozaki, 2008). Cyanobacteria constitute a broad group of Gram-negative prokaryotes with the ability to perform oxygenic photosynthesis in a similar fashion as higher plants (Stanier and Cohen-Bazire, 1977; Woese, 1987). They are considered the main organisms responsible for the ‘Great Oxidation Event’, transforming the atmosphere from a primordial reduced state (with no O_2_ available) to the oxidative atmosphere (with free O_2_) that we have in the present day (Lyons et al., 2014). These photosynthetic organisms live in a wide variety of environments, such as freshwater or marine ecosystems, and play a crucial role on primary biomass production, carbon, and nitrogen cycle (Fuchsman et al., 2019).

**Figure 1:**
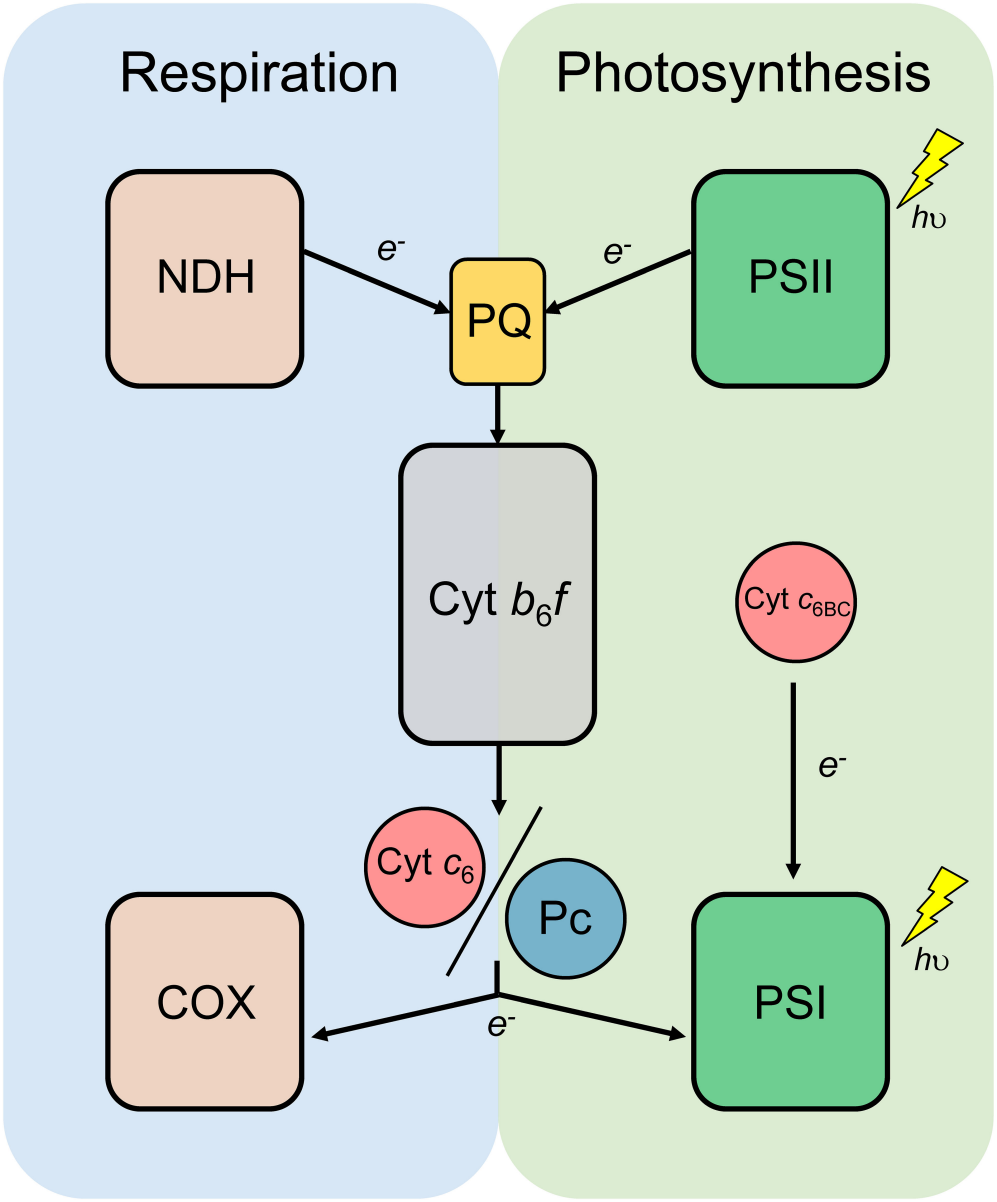
Schematic diagram of the photosynthetic and respiratory electron transport chain of cyanobacteria. Cytochrome *b*_6_ *f* complex (Cyt *b*_6_ *f*) and the redox transporters plastoquinone (PQ), plastocyanin (Pc) and Cytochrome *c*_6_ (Cyt *c*_6_) are shared by both photosynthesis and respiration. NADH dehydrogenase (NDH); Photosystem II (PSII); Photosystem I (PSI); Cytochrome *c* oxidase (COX); Cytochrome *c*_6B_ (Cyt *c*_6BC_).

In cyanobacteria, both photosynthetic and respiratory electron transport chains are present in the same membrane systems (**Figure 1**), sharing some key elements, such as cytochrome (Cyt) *b*_6_ *f* complex and soluble electron carriers plastocyanin (Pc) and Cyt *c*_6_ (Mullineaux, 2014). Both soluble electron carriers are present in a great number of cyanobacteria and green algae and can perform the same function, oxidising Cyt *f* and donating electrons to photosystem (PS) I, in photosynthesis, or to Cyt *c* oxidase (COX), in respiration (Hervás et al., 2003). However, throughout evolution Pc has replaced Cyt *c*_6_, and therefore plants only produce Pc, and, in some cases, a cryptic version of Cyt *c*_6_, Cyt *c*_6A_, the function of which is still unknown (Molina-Heredia et al., 2003; Worrall et al., 2008). Cyt *c*_6_ is present in all sequenced cyanobacteria (Ki, 2005). Besides, most of the cyanobacteria also contain other homologues to Cyt *c*_6_ (Ki, 2005; Bialek et al., 2008). These Cyt *c*_6_-like proteins have been classified as Cyt *c*_6B_ or Cyt *c*_6C_, according to their resemblance to Cyt *c*_6A_ from plants or Cyt *c*_6_ from cyanobacteria, respectively (Bialek et al., 2008). In a recent study, Slater and collaborators (2021) proposed the common origin of both Cyt *c*_6B_ and Cyt *c*_6C_, being Cyt *c*_6B_ a paralogous of Cyt *c*_6_ and Cyt *c*_6C_ an orthologue of Cyt *c*_6B_, with the suggested name of Cyt *c*_6BC_. However, this study does not cover the presence of a third Cyt *c*_6_-like protein that is homologous to Cyt *c*_6_, the so-called Cyt *c*_6-3_ (Torrado et al., 2017). In that study, the newly discovered Cyt *c*_6_-like protein was characterised, and it was found that its redox potential was closer to Cyt *c*_6_ (+300 mV). Members of the Cyt *c*_6BC_ branch have shown to have a less positive redox potential (around +150-200 mV), which will make them unable to physiologically oxidise Cyt *f*, the main electron donor to Cyt *c*_6_ (Molina-Heredia et al., 2003; Bialek et al., 2008; Reyes-Sosa et al., 2011; Bialek et al., 2014). Furthermore, the specific residue in the position 61, which is a tyrosine conserved in Cyt *c*_6BC_ branch, was not conserved neither in Cyt *c*_6_ nor in Cyt *c*_6-3_, which discarded Cyt *c*_6-3_ as a possible member of Cyt *c*_6BC_ group. The present study expands the current understanding of the Cyt *c*_6_-like proteins by reconciling the recently discovered Cyt *c*_6-3_, with the proposed name of Cyt *c*_6D_. We support our statement with novel physiological data of these Cyt *c*_6_-like proteins in photosynthesis and respiration and shed light on the possible function of these proteins homologous to Cyt *c*_6_.

## Materials and methods

### Bacterial strains and culture conditions

*Nostoc* sp. PCC 7119 (ATCC 29151) (Adolph and Haselkorn, 1971) was grown in BG11 (Rippka et al., 1979) (containing NaNO_3_ as N source) or BG11_0_ (lacking any source of combined nitrogen) medium at 30 °C under standard light conditions (25 μmol photons m^-2^·s^-1^ from led white lamps). Cultures were maintained in an orbital shaker (100 rpm) during liquid experiments or in solidified medium (1% w/v Difco Agar). For selection of mutant strains, Streptomycin and Spectinomycin antibiotics were added to the media at a final concentration of 5 μg·mL^-1^ each as described previously (Torrado et al., 2019). Heterocyst formation was induced by transferring cultures grown in BG11 to BG11_0_, after centrifugation at 3000 ×*g* and washing of the cultures with BG11_0_.

### Protein purification and membrane preparation procedures

Cyt *c*_6_, *c*_6BC_, *c*_6D_ and *f* were expressed and purified as described previously (Molina-Heredia et al., 1998; Molina-Heredia et al., 1999; Molina-Heredia et al., 2001; Albarrán et al., 2005; Reyes-Sosa et al., 2011; Torrado et al., 2017). In short, *Escherichia coli* strains were co-transformed with the plasmids bearing each Cyt and the support plasmid pEC86, which encodes the *E. coli* genes required for Cyt *c* maturation (Arslan et al., 1998), in LB medium supplemented with the corresponding antibiotic at 37 °C during 24 h with continuous shaking (300 rpm). After the incubation, proteins were extracted from the periplasmic fraction after three freeze-thawing cycles and subsequent purification in an ion exchange chromatography column. The fractions containing Cyt were followed and analysed spectrophotometrically and in case that the purity was inadequate, another step of chromatography was applied. The A_280_/A_55X_ absorbance ratio was used to estimate the purity of the sample, whereas Cyt *c*_6_, *c*_6BC_, *c*_6D_ and *f* had a maximum absorption peak of the reduced α-band of 553, 553, 552 and 556, respectively. Isolated heterocysts were purified as described in Torrado et al., 2019. Vegetative and heterocyst membranes enriched in terminal oxidases were purified as described previously (Schmetterer et al., 2001; Torrado et al., 2019). In short, isolated membranes from *Nostoc* sp. PCC 7119 were prepared from 800 mL of cultures at a cellular density of 3-6 µg of Chl *a*·mL^-1^. Cells were centrifuged at 12,000 ×*g* for 5 minutes, resuspended in 10 ml HEPES buffer (10 mM HEPES pH 7.4 and 6 mM NaCl) with supplementation of 20% sucrose (w/v) and 10 mg of lysozyme. The suspension was incubated 30 minutes at 37°C and centrifuged at 12,000 ×*g* for 5 minutes. The pellet was resuspended in 10 mL of ice-cold HEPES buffer and incubated on ice for 1 hour. The suspension was supplemented with 1 mM phenylmethylsulfonyl fluoride and 0.005% (w/v) DNase I and passed through a French press three times at 11,000 psi. After this, the suspension was centrifuged at 4°C at 12,000 ×*g* for 10 minutes, resuspended in 5 mL of ice-cold HEPES buffer and homogenizer in a Potter, to a final concentration of 3-5 mg·mL^-1^ of total protein.

### Isothermal titration calorimetry experiments

Cyt samples for isothermal titration calorimetry (ITC) were oxidised (Cyt *c*_6_, *c*_6BC_ and *c*_6D_) or reduced (Cyt *f*) with 10 µM potassium ferrocyanide or 5 mM sodium ascorbate, respectively. Subsequently, samples were extensively dialysed with 5 mM of phosphate buffer (pH 7.0) to remove the oxidising/reducing agents. ITC experiments were carried out in 5 mM phosphate buffer, pH 7.5, supplemented with 0.02% (w/v) Triton X-100, using an Auto-iTC200 instrument (MicroCal, Malvern-Panalytical) at 25 °C and a stirring speed of 1000 rpm. The reference cell was filled with distilled water. The experiments were carried on through successive additions (2 μL injections) of concentrated Cyts *c*_6_, *c*_6BC_ and *c*_6D_ proteins (400 µM) to the sample cell containing the binding partner Cyt *f* (40 µM). All solutions were degassed before titrations. Titrant was injected at appropriate time intervals to ensure that the thermal power signal returned to the baseline prior to the next injection. Control experiments of the dilution of individual injected proteins were conducted and these reference heat values were subtracted from measuring values of test titrations when appropriate. The binding isotherm (ligand-injected normalized heat per injection as a function of the molar ratio) was analysed with Origin 7 (OriginLab). In all cases the heat evolved during titrations could be well fitted to a 1:1 binding stoichiometry, and the association constant, K_A_ (and the dissociation constant, K_D_), and the binding enthalpy (ΔH) and entropy (ΔS) values for the interaction process were estimated (Velázquez-Campoy et al., 2004). Estimated errors in the determined values were 15% for the equilibrium constants, 5% for the binding enthalpy and entropy, and 2% for the binding Gibbs energy.

### Molecular docking

The ClusPro online server (Comeau et al., 2004, https://cluspro.bu.edu/queue.php) was used to perform the protein-protein docking. Model zero was selected out of the top ten models in the balanced order. The structural model was created and visualised using Chimera 1.11 (Pettersen et al., 2004) with colours applied to label the two proteins and their heme groups to facilitate visualization and interpretation.

### Phylogenetic analysis

Multiple sequence alignment was built using ClustalW using 152, 149 and 86 sequences identified as Cyt *c*_6_, Cyt *c*_6BC_ or Cyt *c*_6D_, respectively (**Supplementary Table 1**). Sequence identification was performed using the CyanoOmicsDB database (Zhou et al., 2021). Genome comparison analysis performed using the bioinformatics tools available on the online platform Biocyc (Karp et al., 2019; Karp et al., 2020). Phylogenetic tree was constructed by maximum likelihood method with Geneious version 2023.0 created by Biomatters (https://www.geneious.com), inferred using a neighbour-joining algorithm and Jukes-Cantor as the genetic distance model, using the Bootstrap (n = 100) method (Felsenstein, 1985).

### Oxygen-uptake measurement

Rates of respiration were assessed by measuring the consumption of O_2_ over time in an Oxygraph O_2_ electrode (Hansatech, Cambridge, UK) in a double-jacket thermoregulated glass vessel as described previously (Torrado et al., 2019). The reaction mixture contained, in a final volume of 1 mL, 5 mM of HEPES buffer (pH 7.5), 2.5 mM of NaCl, 2 mM of sodium ascorbate, COX-enriched membranes equivalent to 3-5 mg·mL^-1^ of total protein and 20 µM of Cyt *c*_6_, Cyt *c*_6B_ or Cyt *c*_6D_.

## Results and discussion

### Phylogenetic approach to accommodate the recently discovered Cyt *c*_6D_ group

The phylogeny of Cyt *c*_6_-like proteins has been the subject of study for the past 15 years (Bialek et al., 2008; Reyes-Sosa et al., 2011; Bialek et al., 2014; Howe et al., 2016; Torrado et al., 2016; Torrado et al., 2017; Slater et al., 2021). Bialek et al. (2008) performed a comparative study of the sequence of Cyt *c*_6_ and Cyt *c*_6_-like proteins, concluding that Cyt *c*_6_-like proteins constitutes a new branch separated from Cyt *c*_6_. In addition, they divided them into two groups which they called Cyt *c*_6B_ and Cyt *c*_6C_ based on a phylogenetic analysis. A new analysis performed by Slater et al. (2021) including a higher number of sequences showed that the distinction between Cyt *c*_6B_ and Cyt *c*_6C_ can occur by taxon sampling rather than by differences in function. The similarity of crystal structures, surface electrostatic potential distribution, and midpoint redox potentials of proteins from both B and C subgroups, points to the fact that Cyt *c*_6B_ and Cyt *c*_6C_ could perform a similar function and are, probably, orthologs (Zatwarnicki et al., 2014; Slater et al., 2021). However, a group of Cyt *c*_6_ homologous that was first found in heterocyst-forming filamentous cyanobacteria, named as Cyt *c*_6-3_ (Torrado et al., 2017), has not been integrated in the current phylogeny. We are aware that the nomenclature of these Cyt *c*_6_-like proteins can result confusing, but because the function of these Cyt *c*_6_-like proteins is not elucidated, a clear nomenclature cannot be established. For now, we propose to keep the former nomenclature, whereas the first Cyt *c*_6_-like protein found in cyanobacteria will be Cyt *c*_6BC_ and the second one, the formerly called Cyt *c*_6-3_ which only appears in filamentous cyanobacteria, Cyt *c*_6D_.

**Figure 2:**
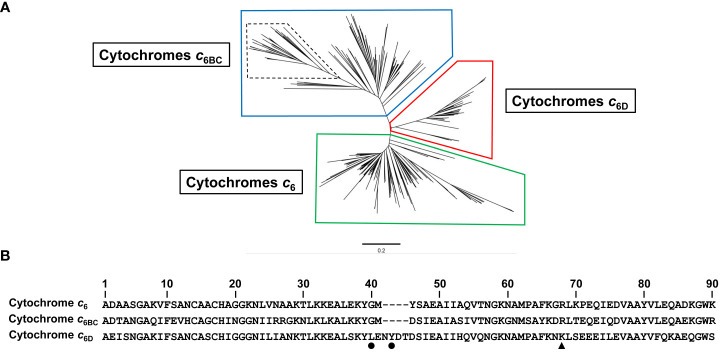
Sequence analysis of 387 sequences identified as Cyt *c*_6_, Cyt *c*_6BC_ or Cyt *c*_6D_. **(A)** Phylogenetic tree of soluble cytochromes described in cyanobacteria using Neighbour-Joining clustering method. In boxes, clustered sequences from Cyt *c*_6_ (Green), Cyt *c*_6BC_ (Blue) and Cyt *c*_6D_ group (Red). Dashed lines inside Cyt *c*_6BC_ group highlight the formerly classified Cyt *c*_6B_ proteins subgroup, while the rest of the sequences of that group were classified as Cyt *c*_6C_ proteins subgroup. **(B)** Consensus sequence of Cyt *c*_6_, Cyt *c*_6BC_ and Cyt *c*_6D_, using 152, 149 and 86 sequences of each protein, respectively, using ClustalW alignment for each group of sequences. Black dots point to the two key residues conserved only in Cyt *c*_6D_ group. Black triangle points to the conserved arginine crucial to the interaction with PS I in Cyt *c*_6_ and Cyt *c*_6BC_. Sequences are numbered according to the longest consensus sequence which is Cyt *c*_6D_. The bar represents 0.2 substitutions per site.

To investigate the distribution of Cyt *c*_6_ of cyanobacteria and to include the presence of this new group, we mapped a phylogenetic tree of cyanobacterial sequences of Cyt *c*_6_-like proteins including the new group of Cyt *c*_6D_ (**Figure 2A**). The phylogenetic analysis was based on 387 sequences of Cyt *c*_6_, Cyt *c*_6BC_ and Cyt *c*_6D_ found in cyanobacteria from public databases. The sequences were aligned using ClustalW method and a phylogenetic tree was inferred using maximum-likelihood method (see Material and Methods). The results (**Figure 2A**) showed that the newly classified Cyt *c*_6D_ forms an independent group clearly separated from Cyt *c*_6_ or Cyt *c*_6BC_. In the previous publication in which Cyt *c*_6D_ was described (Torrado et al., 2017), where the genomes and tools were more limited, Cyt *c*_6D_ was found only in heterocyst-forming filamentous cyanobacteria. A more comprehensive study involving a higher number of Cyt *c*_6_ protein sequences has revealed that Cyt *c*_6D_ is present in all type of filamentous cyanobacteria, independently of the heterocyst formation, but it is not present in unicellular cyanobacteria. Besides, this sequence analysis has revealed a conserved sequence pattern that is present in all Cyt *c*_6D_ (**Figure 2B**). The conserved pattern has been identified as L-X-X-Y and starts at position 40 of the consensus sequence (most common amino acid at that position; Liljas, 2013) of Cyt *c*_6D_. Also, in Cyt *c*_6_ and in Pc appears a single arginine residue at position 64 in Cyt *c*_6_ (**Figure 2B**) that is strictly conserved in both proteins and results crucial for their interaction with PS I (Molina-Heredia et al., 2001). This residue is also strictly conserved in the position 64 in Cyt *c*_6BC_, but it has been replaced by lysine (position 68, black triangle in **Figure 2B** **)** in Cyt *c*_6D_. In *Nostoc*, we have previously reported that Cyt *c*_6BC_ can react with PS I, but in a less efficient manner than Cyt *c*_6_. (Reyes-Sosa et al., 2011). However, Cyt *c*_6D_ does not react with PS I (Torrado et al., 2017).

**Figure 3:**
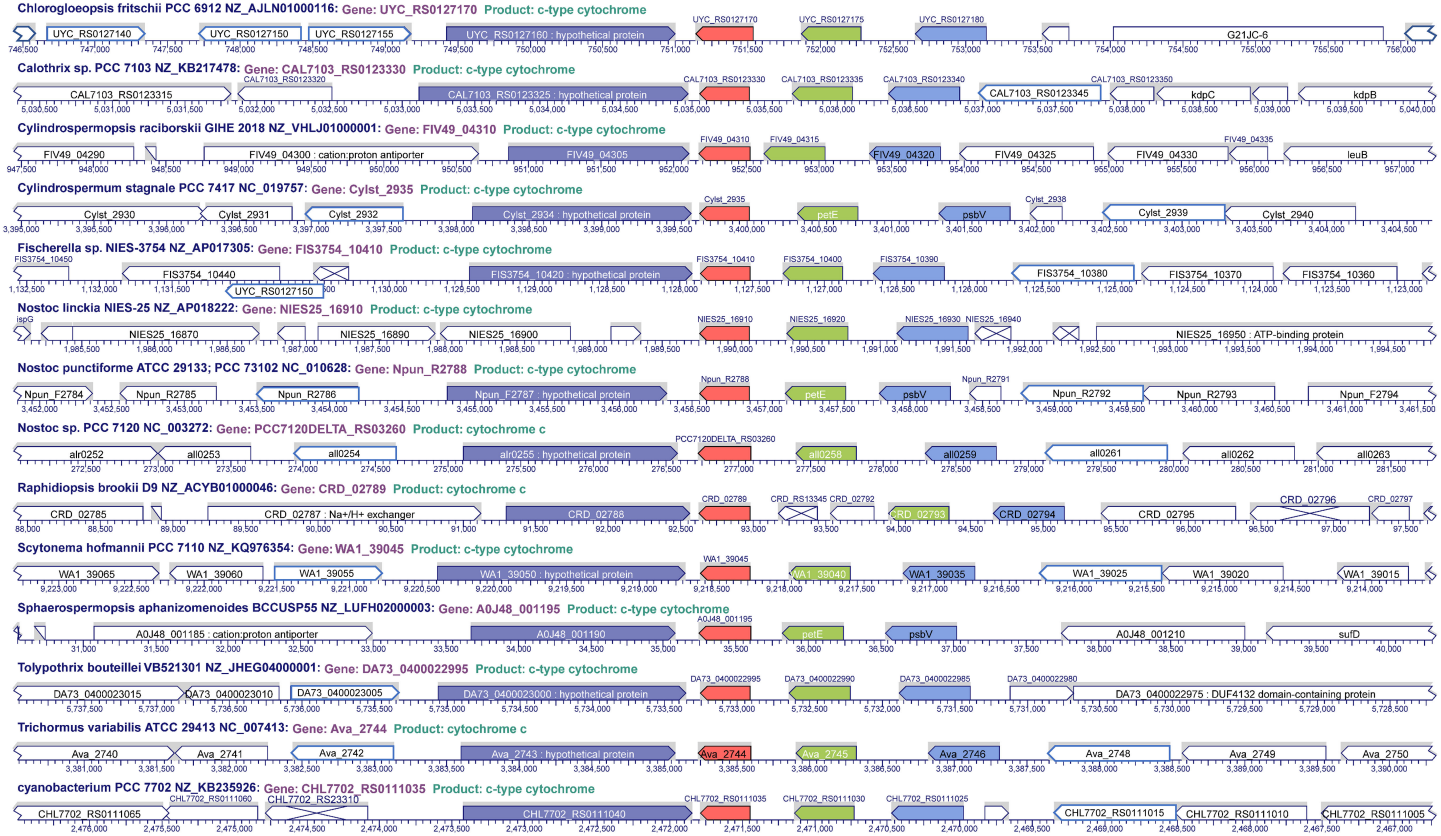
Genome localisation of Cyt *c*_6D_ in filamentous cyanobacteria. In red, *pet*J-3 (Cyt *c*_6D_); in green, *pet*E (Plastocyanin); in pale blue, *psb*V (Cyt *c*_550_); in dark blue, hypothetical protein conserved upstream *pet*J-3.

An interesting feature found in this analysis was the position and distribution of Cyt *c*_6D_ within cyanobacteria. We analysed the genomic context of the gene encoding Cyt *c*_6D_ in filamentous cyanobacteria, finding that the position and distribution of the gene in the genome seems to follow a specific pattern in most of the studied organisms (**Figure 3**, **Supplementary Table 1**). Gene encoding Cyt *c*_6D_ is located in a conserved region, whereas the genes *psbV* (Cyt *c*_550_), *petE* (Pc) and *petJ-3* (Cyt *c*_6D_) are located in the same order and direction of the expression (**Figure 3**), in contraposition to the positions of *petJ* (Cyt *c*_6_) or *petJ-2* (Cyt *c*_6B_) which are unevenly distributed (**Supplementary Figure 1**). Cyt *c*_550_ is a component of the PS II complex in cyanobacteria and some eukaryotic algae, such as red and brown algae (Roncel et al., 2012), and Pc acts as soluble electron carrier between Cyt *b*_6_ *f* complex and PS I. The fact that the gene that codes for Cyt *c*_6D_ is in the same cluster as the genes that code for Cyt *c*_550_ and Pc may lead us to think that Cyt *c*_6D_ function is related to the photosynthetic electron transport chain. However, this function should be different from Cyt *c*_6_ and Pc, because as we mentioned before, Cyt *c*_6D_ is not able to reduce PS I (Torrado et al., 2017). Another gene is frequently found downstream of Cyt *c*_6D_, a hypothetical protein with partial similarity to *Ton*B (BlastP), but no further information is provided in this study (**Figure 3**).

### Analysis of the interaction of Cyt *c*_6BC_ and Cyt *c*_6D_ with Cyt *b*_6_ *f* complex

We investigated whether Cyt *c*_6BC_ and Cyt *c*_6D_ can interact with the subunit Cyt *f* of Cyt *b*_6_ *f* complex. One of the key factors for this interaction is the redox potential of the Cyts. As we discussed before, the redox potential of Cyt *c*_6BC_ (+199 mV) and Cyt *c*_6D_ (+300 mV) is lower than that of Cyt *c*_6_ (+335 mV), which at first glance indicates that this interaction is unlikely to happen (Molina-Heredia et al., 1998; Reyes-Sosa et al., 2011; Torrado et al., 2017). However, in our previous study (Torrado et al., 2017), we analysed the redox potential under the physiological condition of thylakoidal lumen of pH 4, instead of the standard pH 7. We found that the redox potential of Cyt *c*_6D_ (+343 mV) became isopotential with Cyt *c*_6_ (+340 mV) under that condition. Still, Cyt *c*_6BC_ (+230 mV) was found to be far from the redox potential of the other Cyts. However, we could not measure the empirical redox potential of soluble Cyt *f in vitro* at pH 4, as this protein was denatured and degraded at that pH. We explain this phenomenon because physiologically, Cyt *f* should be embedded within Cyt *b*_6_ *f* membrane complex, but in the soluble form heterologously expressed, the integrity of this protein cannot withstand the acidic pH. In summary, with the redox potential found under those conditions, Cyt *c*_6D_ would be able to oxidise Cyt *f* while Cyt *c*_6BC_ will be unable to do it. However, the redox potential is not a sufficient condition to demonstrate that the interaction can happen between the proteins, since it only establishes the direction of the electron transfer in case they would interact. To evaluate if the proteins can interact *in vitro*, we performed Isothermal Titration Calorimetry (ITC) experiments and protein-protein docking analysis.

**Figure 4:**
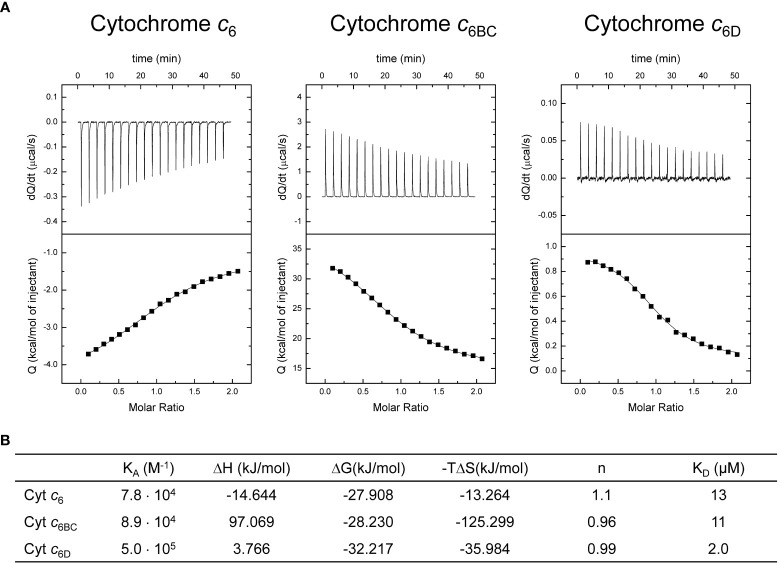
Calorimetric titrations of Cyt *c*_6_, Cyt *c*_6BC_ and Cyt *c*_6D_ corresponding to the formation of the complex with Cyt *f*. **(A)** Thermograms (thermal power as a function of time, upper plots) and binding isotherms (ligand-normalised heat effects per injection as a function of the molar ratio, lower plots). The continuous thin lines correspond to the fits according to the single binding site model. **(B)** Thermodynamic binding parameters for the complexes. K_A_: association constant; ΔH: binding enthalpy; ΔG: binding Gibbs energy; –TΔS: binding entropic contribution; n: binding stoichiometry; K_D_: dissociation constant. Each experiment was performed with three independent replicates.

For ITC experiments we prepared purified samples of each soluble Cyts in an oxidised state, and samples with Cyt *f* completely reduced to facilitate the interaction, at 25 °C. In all the cases, we observed interaction between the proteins, with a 1:1 binding stoichiometry (**Figure 4A**). The analysis of the binding isotherms, according to a ligand-binding model with a single binding site, allowed us to determine the thermodynamic interaction parameters (**Figure 4B**). Both, K_A_ and K_D_ for the interaction of Cyt *c*_6BC_ with Cyt *f* are similar to those obtained with Cyt *c*_6_. However, K_A_ and K_D_ for the interaction of Cyt *c*_6D_ with Cyt *f* are 6-fold higher and lower, respectively, than those obtained with both Cyt *c*_6_ and Cyt *c*_6BC_. These results show that Cyt *c*_6D_ has a higher affinity for Cyt *f* than Cyt *c*_6_. Looking at the thermodynamic parameters, although the ΔG values of the interaction of the three Cyts are somewhat similar, the values ​​of ΔH and –TΔS indicate that the three interactions are quite different. In the case of Cyt *c*_6_, whose isoelectric point (p*I*) is 9.0 (Molina-Heredia et al., 1998), both ΔH and –TΔS are negative, indicating that the interaction with Cyt *f* might be driven by attractive electrostatic interactions and by hydrophobic interactions. This agrees with the fact that Cyt *c*_6_ presents electrostatic and hydrophobic regions interacting with both PS I and Cyt *f* (Molina-Heredia et al., 1999; Crowley et al., 2002; Albarrán et al., 2005). In the case of Cyt *c*_6BC_, ΔH and –TΔS are large and of opposite sign, cancelling partially each other. These values ​​indicate that, although an electrostatic repulsion may occur, the interaction is exergonic and might be driven primarily by hydrophobic interactions. This is in agreement with the data previously reported (Reyes-Sosa et al., 2011) that described the conserved hydrophobic interaction surface with Cyt *f* in Cyt *c*_6BC_; but not the surface of electrostatic interaction, being positive in Cyt *c*_6_, but negative in Cyt *c*_6BC_, even though it has a p*I* of 8.0, very close to that of Cyt *c*_6_. Likewise, in the case of Cyt *c_6_*_D_, whose p*I* is 5.2 (Torrado et al., 2017), the interaction with Cyt *f* is driven by hydrophobic forces, not presenting attractive electrostatic interactions. As in the case of Cyt *c*_6BC_, Cyt *c*_6D_ preserves the hydrophobic surface for the interaction with Cyt *f*; however, the region for the electrostatic interaction that is present in Cyt *c*_6_ results practically neutral in Cyt *c*_6D_ (Torrado et al., 2017).

**Figure 5:**
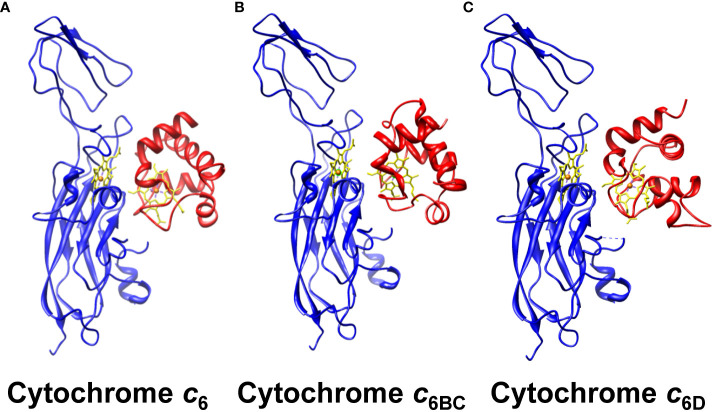
Molecular docking of the interaction between of the soluble Cyt *c*_6_-like proteins and Cyt *f*. The molecular interaction of Cyt *f* with **(A)** Cyt *c*_6_, **(B)** Cyt *c*_6BC_ and **(C)** Cyt *c*_6D_ is represented, with the heme groups aligned. Cyt *f* from Cyt *b*_6_ *f* complex is shown in blue; soluble Cyts are shown in red; heme groups are shown in yellow, with the characteristic iron in orange. The equilibrated models of minimum energy were selected for each case, using the online server Cluspro V.2.0. The PDB data with the structures used can be found in the supplementary data.

Besides the fact that the interaction can happen between Cyt *c*_6_ paralogous proteins and Cyt *f*, one of the key factors for a redox interaction involving cytochromes lays in the orientation of their heme groups (Cruz-Gallardo et al., 2012). The interaction may be happening but the heme groups might be so distant that the electron transfer would not be possible. To assess such orientation along the interaction, we performed a structural modelling by molecular docking of the soluble Cyts with Cyt *f* (**Figure 5**). As a control, we used Cyt *c*_6_, considering that its interaction and orientation with Cyt *f* has been previously reported (Díaz-Moreno et al., 2005). The molecular docking was conducted using the Cluspro V.2.0 server, selecting the equilibrated models with minimum energy. According to these models, Cyt *c*_6BC_ and Cyt *c*_6D_ (**Figures 5B, C**) will interact with Cyt *f* through the same hydrophobic area as Cyt *c*_6_ (**Figure 5A**). Further, their heme groups will be oriented opposed to the Cyt *f* heme group, as it happens as well with Cyt *c*_6_. In conclusion, Cyt *c*_6BC_ and Cyt *c*_6D_ could interact with Cyt *f* in the same areas, with the same orientation as Cyt *c*_6_, and with a distance between the redox centres similar to that of Cyt *c*_6_, which would allow an electron transfer between them.

### Functional analysis of the interaction of Cyt *c*_6BC_ and Cyt *c*_6D_ with cytochrome *c* oxidase in respiration

**Figure 6:**
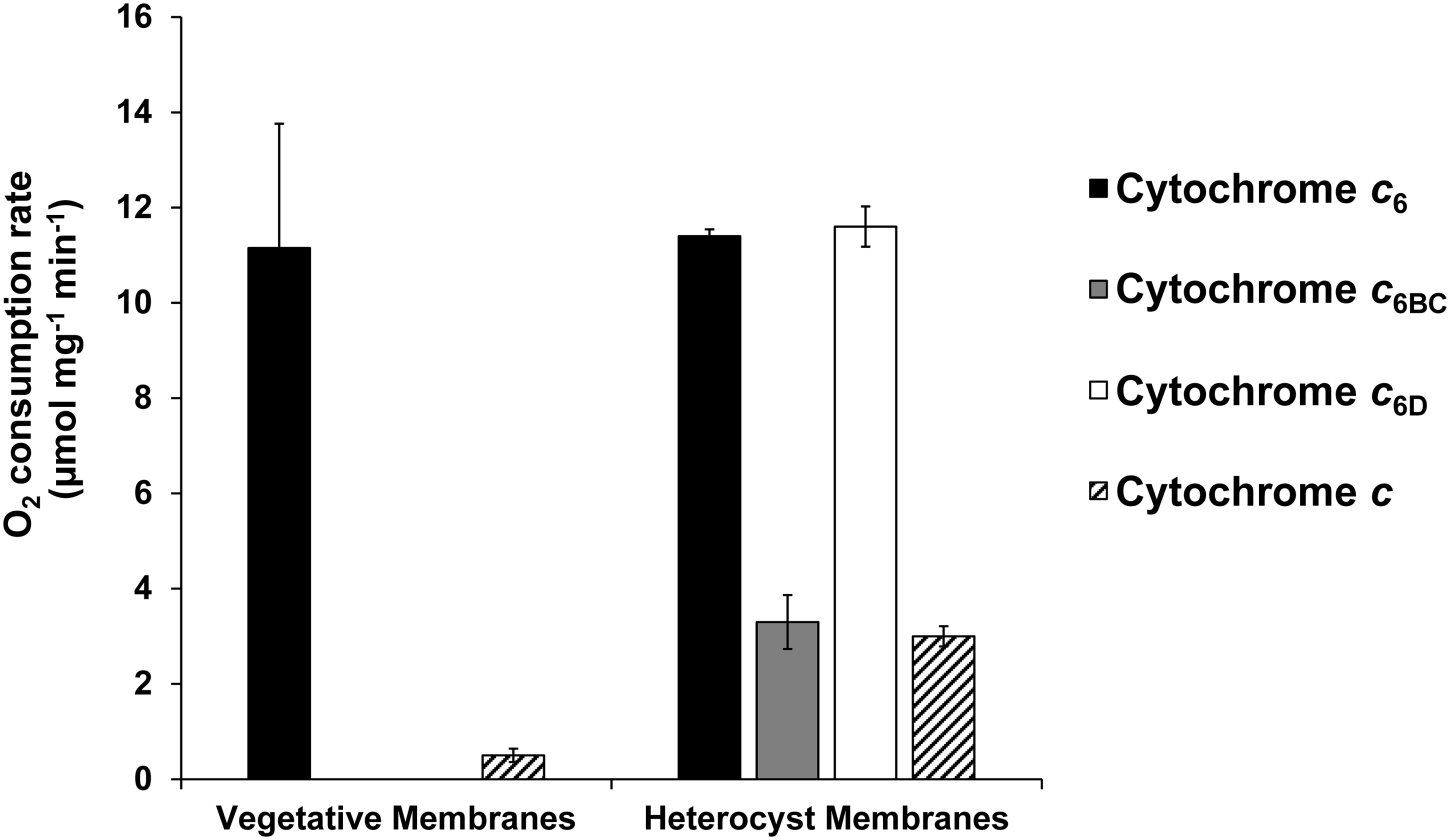
Oxygen uptake rate of Cyt *c*_6_, Cyt *c*_6BC_ and Cyt *c*_6D_ with isolated membranes enriched in terminal oxidases of *Nostoc* sp. PCC 7119. Rates of oxygen consumption were measured in the presence of Cyt *c*_6_ (black), Cyt *c*_6BC_ (grey), Cyt *c*_6D_ (white) and Cyt *c* from horse (striped) in either vegetative or heterocyst cell membranes. Oxygen consumption rate was calculated per mg of membrane protein of the sample. Cyt *c* was used as a control for unspecific interaction of a Cyt *c*-type protein. Vegetative membranes: membranes obtained from vegetative cells, cultured in continuous light and with combined nitrogen. Heterocyst membranes: membranes obtained from isolated heterocyst, cultured in continuous light and without combined nitrogen. Plotted bars are the average from three independent replicates; error bars represent standard deviations from the mean.

One of the key questions yet to be addressed is the role of these Cyt *c*_6_-like proteins in respiration. As we described before, Cyt *c*_6_ and Pc are the main interaction partners with terminal oxidases in respiration. However, little information has been provided about the interaction of Cyt *c*_6_-like proteins in respiration (Torrado et al., 2016). *Nostoc* sp. PCC 7119 is an heterocyst forming filamentous cyanobacterium that can express two different set of *aa3*-type copper oxidases. One of these oxidases is expressed in vegetative cells and is the main respiratory oxidase, also called COX1 (Valladares et al., 2014). In heterocyst, which are differentiated cells under nitrogen-deficient conditions, COX2, the alternative *aa3*-type oxidase, is expressed. Thus, under nitrogen-deficient conditions where vegetative cells and heterocysts cohabit in the filament, COX1 is expressed only in vegetative cells and COX2 is expressed only in heterocysts. As we described before, Cyt *c*_6D_ gene is only found in filamentous cyanobacteria, including heterocyst-forming cyanobacteria. Hence, our approach comprised the study of both terminal oxidases, with particular interest on the heterocyst-specific COX2 (Valladares et al., 2014). To address this question, we performed O_2_ uptake experiments with membranes enriched in terminal oxidases in the dark (**Figure 6**), using an oxygen electrode (Oxygraph, Hansatech). First, we evaluated the reactivity with the main oxidase that is expressed only in vegetative cells, COX1. In this condition, membranes enriched in COX1 were isolated and assessed against the three Cyts. As expected, Cyt *c*_6_ reacts with these membranes producing a high rate of oxygen consumption. However, neither Cyt *c*_6BC_ or Cyt *c*_6D_ produced any significant increase in oxygen consumption, indicating no reaction with COX1. Next, we purified heterocysts, and from these preparations, we isolated membranes enriched in COX2. In this condition, we found that Cyt *c*_6_ behaved as described previously, reacting with COX2 and producing an elevated oxygen consumption (Torrado et al., 2019). However, Cyt *c*_6BC_ was not able to react with these membranes enriched in COX2, observing only an unspecific oxygen consumption, at the level of a non-native Cyt *c*. Surprisingly, Cyt *c*_6D_ was able to react with membranes enriched in COX2 with a similar rate of oxygen consumption as Cyt *c*_6_. Thus, contrary to Cyt *c*_6_, Cyt *c*_6D_ reacts specifically with COX2.

These results could lead us to think that Cyt *c*_6D_ may transport electrons from Cyt *b*_6_ *f* complex to the COX2, specific to heterocysts. However, previous *in vivo* GFP report of the Cyt *c*_6D_ promoter activity experiments showed that in the presence of combined nitrogen, Cyt *c*_6D_ is homogeneously expressed in all the cells of the filament, and that under nitrogen starvation, the expression of Cyt *c*_6D_ was partially repressed in vegetative cells, but such repression was practically complete in heterocysts (Torrado et al., 2017).

## Conclusions

**Figure 7:**
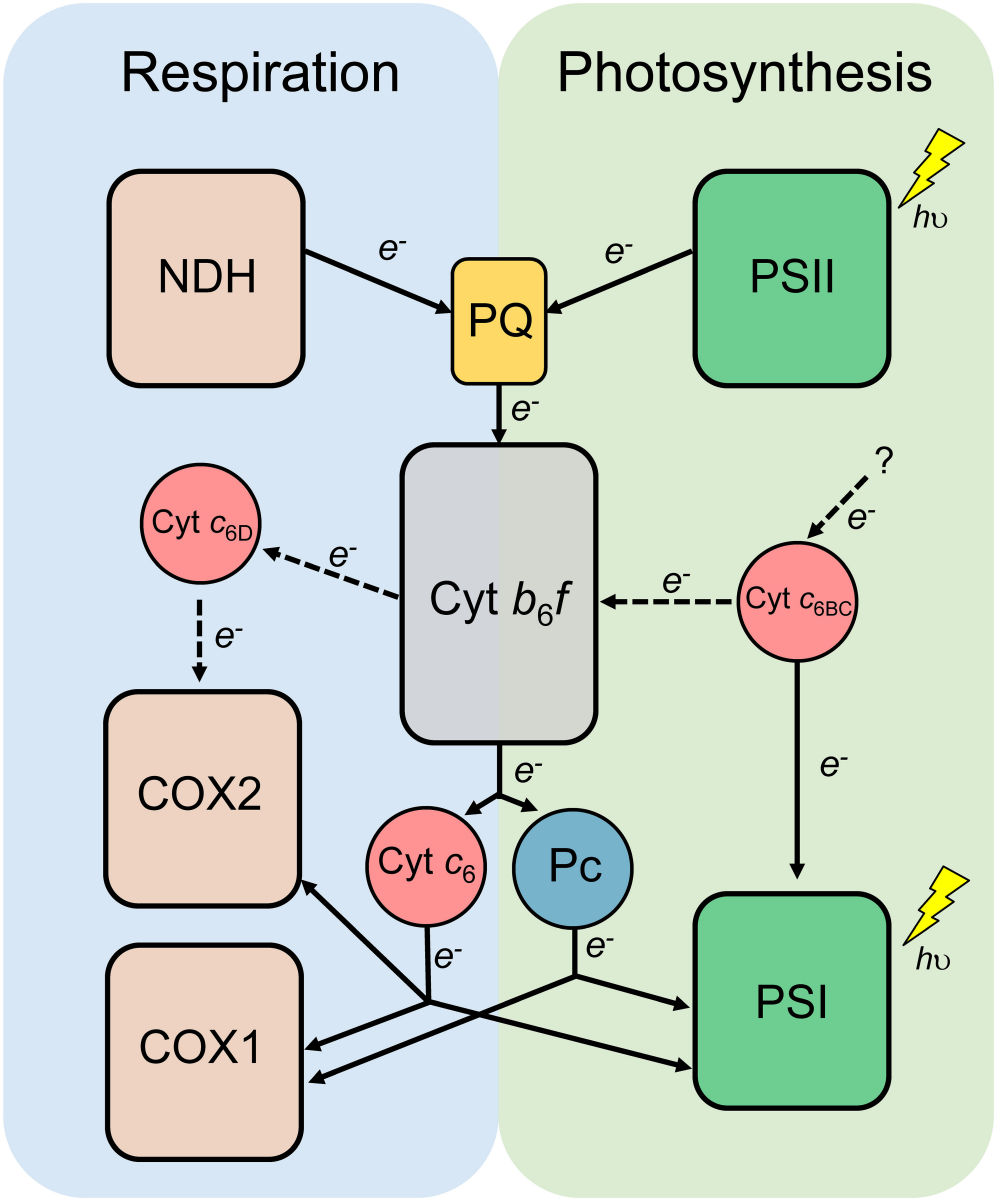
Proposed model of the Cyt *c*_6_-like proteins in photosynthetic and respiratory electron transport chain of heterocyst-forming cyanobacteria. Dashed lines represent the new findings of this study. Cytochrome *b*_6_ *f* complex (Cyt *b*_6_ *f*) and the redox transporters plastoquinone (PQ), plastocyanin (Pc) and Cytochrome *c*_6_ (Cyt *c*_6_) are shared by both photosynthesis and respiration. NADH dehydrogenase (NDH); Photosystem II (PSII); Photosystem I (PSI); Cytochrome *c* oxidase 1 specific of vegetative cells (COX1); Cytochrome *c* oxidase 2 specific of heterocysts (COX2); Cytochrome *c*_6B_ (Cyt *c*_6BC_); Cytochrome *c*_6D_ (Cyt *c*_6D_).

In summary, the present work expands the current knowledge of Cyt *c*_6_-like proteins by the inclusion in the phylogeny of the recently discovered group Cyt *c*_6-3_, renamed in this work as Cyt *c*_6D_. We have provided information about the differences between Cyt *c*_6BC_ and Cyt *c*_6D_, being two clearly independent groups of Cyt *c*_6_-like proteins (**Figure 2A**). Also, Cyt *c*_6D_ seems to follow a distribution pattern along the genome of filamentous cyanobacteria that is well-conserved and should be further investigated (**Figure 3**). The differences between these Cyts are not only at sequence level, but also at their physico-chemical properties. The redox potential of both proteins has proved to be significantly different, being Cyt *c*_6D_ isopotential with Cyt *c*_6_ at physiological pH conditions, while Cyt *c*_6BC_ is a less positive redox potential protein in comparison (Torrado et al., 2017). A closer look at their interaction with the Cyt *c*_6_ partner Cyt *f*, both were found to be able to interact with it at protein-protein level (**Figure 4**). Further, the molecular docking revealed that the binding location and orientation of both proteins were adequate for the electron transfer (**Figure 5**). However, as we have stated before, the low redox potential of Cyt *c*_6BC_ will make the electron transfer from Cyt *f* unlikely, from a thermodynamic point of view, (Reyes-Sosa et al., 2011). As the PS I interaction with both proteins has been already tested (Reyes-Sosa et al., 2011; Torrado et al., 2017), we decided to investigate the interaction of these proteins in the respiratory electron transport chain (**Figure 6**). In these experiments, Cyt *c*_6BC_ was proven to not react specifically with either both terminal oxidases of the cyanobacterium. However, Cyt *c*_6D_ could react specifically, with a similar kinetics as Cyt *c*_6_, with the oxidase specific from heterocyst, COX2. These findings open the door to a functional differentiation of Cyt *c*_6_-like proteins (**Figure 7**). A possible function of Cyt *c*_6BC_ could be to connect another redox process with photosynthetic and respiratory electron transport chains, introducing the electrons at the level of Cyt *f* and PS I. On the other hand, Cyt *c*_6D_ appears only in filamentous cyanobacteria. Interestingly, when it is present in the genome, the gene that codes for it is always found in the same cluster as the one that codes for Pc and for Cyt *c*_550_, both of which participate in photosynthesis. However, Cyt *c*_6D_ is not able to react with either PS I or COX1, the specific oxidase of vegetative cells. Although it does efficiently reduce COX2, which is the heterocyst-specific oxidase, its expression is repressed in heterocysts in the standard culture conditions tested (Torrado et al., 2017). However, we cannot rule out that Cyt *c*_6D_ expression could induced under stress conditions or under other culture conditions. Besides, Cyt *c*_6D_ could be capable of interacting with Cyt *f*, showing a higher affinity than Cyt *c*_6_. These results make us think that Cyt *c*_6D_ could be involved in the fine-tuning regulation of electron transport in the vegetative cells of filamentous cyanobacteria, at the level of Cyt *f*.

## Data availability statement

The original contributions presented in the study are included in the article/**Supplementary Files**. Further inquiries can be directed to the corresponding authors.

## Author contributions

AT and FM-H conceived the project. AT, MI-P, AV-C, and FM-H carried out the experiments. AT, VM, CA, and FM-H interpreted the data and discussed the results. AT, VM, and FM-H wrote the manuscript, which was corrected, revised, and approved by all authors.

## Supplementary material

Supplementary Table 1 Sequences used in this study for the analysis and comparison of Cyt *c*_6_-like proteins in cyanobacteria.

Supplementary Figure 1 Schematic of the *pet*J gene localisation in the genome of single cell and filamentous cyanobacteria. Line represents the position in the genome and the direction of the arrow, the direction of the gene expression. The double dash represents different positions within the genome of the same organism. *pet*E: Plastocyanin; *pet*J: Cytochrome *c*_6_; *psb*V: Cytochrome *c*_550_; *pet*J-3: Cytochrome *c*_6D_.
